# Combination of modified carbapenem inactivation method (mCIM) and EDTA-CIM (eCIM) for phenotypic detection of carbapenemase-producing *Enterobacteriaceae*

**DOI:** 10.1186/s12866-020-02010-3

**Published:** 2020-10-17

**Authors:** Ya-Min Tsai, Shining Wang, Hui-Chuan Chiu, Cheng-Yen Kao, Li-Li Wen

**Affiliations:** 1 Department of Clinical Laboratory, En Chu Kong Hospital, No. 399, Fuxing Rd., Sanxia Dist, New Taipei City, 23702 Taiwan; 2 Institute of Microbiology and Immunology, School of Life Science, National Yang-Ming University, No.155, Sec.2, Linong Street, Taipei, 112 Taiwan

**Keywords:** Carbapenemase, *Enterobacteriaceae*, eCIM, mCIM, Phenotypic detection

## Abstract

**Background:**

Carbapenemase-resistant *Enterobacteriaceae* (CRE) cause many serious infections resulting in increasing treatment cost, prolonged hospitalization, and mortality rate. Reduced expression and/or mutations of porins and the presence of carbapenemase promote *Enterobacteriaceae* survival under carbapenem treatments. Development of accurate methods for the detection of antimicrobial resistance is required not only for therapy but also to monitor the spread of resistant bacteria or resistance genes throughout the hospital and community. In this study, we aimed to evaluate the phenotypic methods, Modified Hodge test (MHT), modified carbapenem inactivation method (mCIM), and EDTA-CIM (eCIM) for the detection of carbapenemase-producing *Enterobacteriaceae* (CPE).

**Results:**

The results showed that mCIM had a sensitivity of 100% and a specificity of 100%, whereas the MHT had a sensitivity of 84.8% and a specificity of 97.8% for the 195 CRE isolates tested (105 CPE and 90 non-CPE isolates). The sensitivity of the mCIM/eCIM to detect metallo-carbapenemases in this study was 89.3% and the specificity was 98.7% as compared to the genotypic PCR detection.

**Conclusions:**

These findings indicate that the mCIM combined with eCIM is useful for detecting and distinguishing different types of carbapenemase in *Enterobacteriaceae*.

## Background

*Enterobacteriaceae* are Gram-negative, facultatively anaerobic, non-spore-forming rods and one of the most prevalent causes of invasive infections and nosocomial infections [1]. Infections caused by *Enterobacteriaceae* are often difficult to treat due to the high antimicrobial resistance among clinical isolates [2]. Carbapenems (imipenem, ertapenem, meropenem, and doripenem) are considered as “antibiotics of last-resort” in the treatment of critically ill patients with a variety of bacterial infections due to their broad spectrum among β-lactam antibiotics and relative resistance to hydrolysis by most β-lactamases [3, 4]. However, carbapenem-resistant *Enterobacteriaceae* (CRE) have emerged and become a public health threat worldwide in the past decade [5].

While carbapenems enter bacterial cells via specific porins, the reduced expression and/or mutation of porins plays a critical role in the resistance to carbapenems [6, 7]. Moreover, the presence of carbapenemase genes on conjugative plasmids for the hydrolysis of carbapenems to promote *Enterobacteriaceae* survival under antibiotic treatments is associated with the rapid emergence of CRE [8]. β-lactamases are categorized according to sequence homology into four molecular classes: A, B, C and D [9], whereas carbapenemases are the members of class A, B, and D β-lactamases [10]. Moreover, based on the participation of divalent cations in enzyme activation, carbapenemases are segregated into non-metallo-β-lactamases (zinc-independent serine carbapenemases, classes A and D) and metallo-β-lactamases (MBLs, zinc-dependent, class B) [11]. Therefore, EDTA or dipicolinic acid can serve as chelators to block class B carbapenemases activity by binding zinc [12]. MBL genes such as *bla*_NDM_, *bla*_IMP_, *bla*_VIM_, and non-metallo-carbapenemase genes, *bla*_OXA_ (class D) and *bla*_KPC_ (class A), have been frequently reported in CRE, called carbapenemase-producing *Enterobacteriaceae* (CPE) [8, 13–15].

The characterization of underlying mechanisms leading to carbapenem resistance of clinical isolates is not undertaken by most clinical microbiology laboratories for therapeutic decision-making; however, understanding if an isolate is CPE has significant epidemiological implications for monitoring local epidemiology and also lead to more effective treatment of infections caused by CPE (e.g. ceftazidime-avibactam or meropenem-vaborbactam, which have activity against KPC-producer) [16].

The Modified Hodge test (MHT) is the first Clinical & Laboratory Standards Institute (*CLSI*) recommended growth-based carbapenemase detection test in 2009 with high level of sensitivity and specificity in detecting carbapenemases [17, 18]. Currently, the modified carbapenem inactivation method (mCIM) has been reported to accurately identify carbapenemases but cannot distinguish between serine and metallo-carbapenemases [19, 20]. Therefore, a further modification to mCIM with the addition of EDTA (eCIM) has been endorsed in the CLSI M100-S28 supplement in 2018 to specifically identify metallo-carbapenemases [19, 20]. In this study, we aimed to evaluate the phenotypic detection methods MHT, mCIM, and eCIM for detecting CPE.

## Results

### Detection of carbapenemase genes by PCR

To detect the presence of carbapenemase genes among our 419 CRE isolates, we performed PCR on 7 *Citrobacter koseri*, 8 *Klebsiella aerogenes*, 21 *Enterobacter cloacae*, 73 *E. coli*, and 310 *Klebsiella pneumoniae* isolates. The PCR results showed that 105 (25.1%, 105/419) CRE isolates were genetically characterized to carry carbapenemase genes: *bla*_OXA-48_ was detected in 41 (39.0%) isolates, *bla*_KPC_ was detected in 34 (32.4%) isolates, *bla*_NDM_ in 15 (14.3%) isolates, *bla*_IMP_ in 7 (6.7%) isolates, and *bla*_VIM_ in 3 (2.9%) isolates. Five isolates carried two types of carbapenemase genes (*bla*_NDM_ and *bla*_OXA-48_ in three *E. coli* isolates, and *bla*_KPC_ and *bla*_OXA-48_ in 2 *K. pneumoniae* isolates).

### Phenotypic detection of carbapenemase-producing *Enterobacteriaceae*

The ability of CRE isolates to produce carbapenemases was tested by MHT first. Ninety non-carbapenemase-producing CRE isolates were randomly selected as negative controls. Out of the 195 isolates (105 CPE and 90 non-CPE), 91 were MHT-positive for carbapenemase production (2 false-positive isolates) (Table 1). Moreover, 16 isolates showed false-negative MHT results (Table 1). MHT had excellent sensitivity for the detection of *bla*_OXA-48_ (95.1%) and *bla*_KPC_ (100%), but not *bla*_NDM_ (46.7%), *bla*_IMP_ (57.1%), and *bla*_VIM_ (33.3%) (Table 1). The overall sensitivity and specificity of MHT in this study for the detection of CPE was 84.8 and 97.8%, respectively (Table 1).

**Table 1:** Sensitivity and specificity of Modified Hodge test for the detection of carbapenemase-producing *Enterobacteriaceae*

	OXA-48	KPC	NDM	IMP	VIM	NDM/OXA-48	KPC/OXA-48	carbapenemase (−)	total
TP (n)	39	34	7	4	1	2	2	–	89
FN (n)	2	0	8	3	2	1	0	–	16
TN (n)	–	–	–	–	–	–	–	88	88
FP (n)	–	–	–	–	–	–	–	2	2
Sensitivity (%)^a^	95.1	100	46.7	57.1	33.3	66.7	100	–	84.8
Specificity (%)^b^	–	–	–	–	–	–	–	97.8	97.8

^a^Sensitivity = TP/TP + FN

^b^Specificity = TN/TN + FP

*TP* true positive, *FN* false negative, *TN* true negative, *FP* false positive

**Fig. 1:**
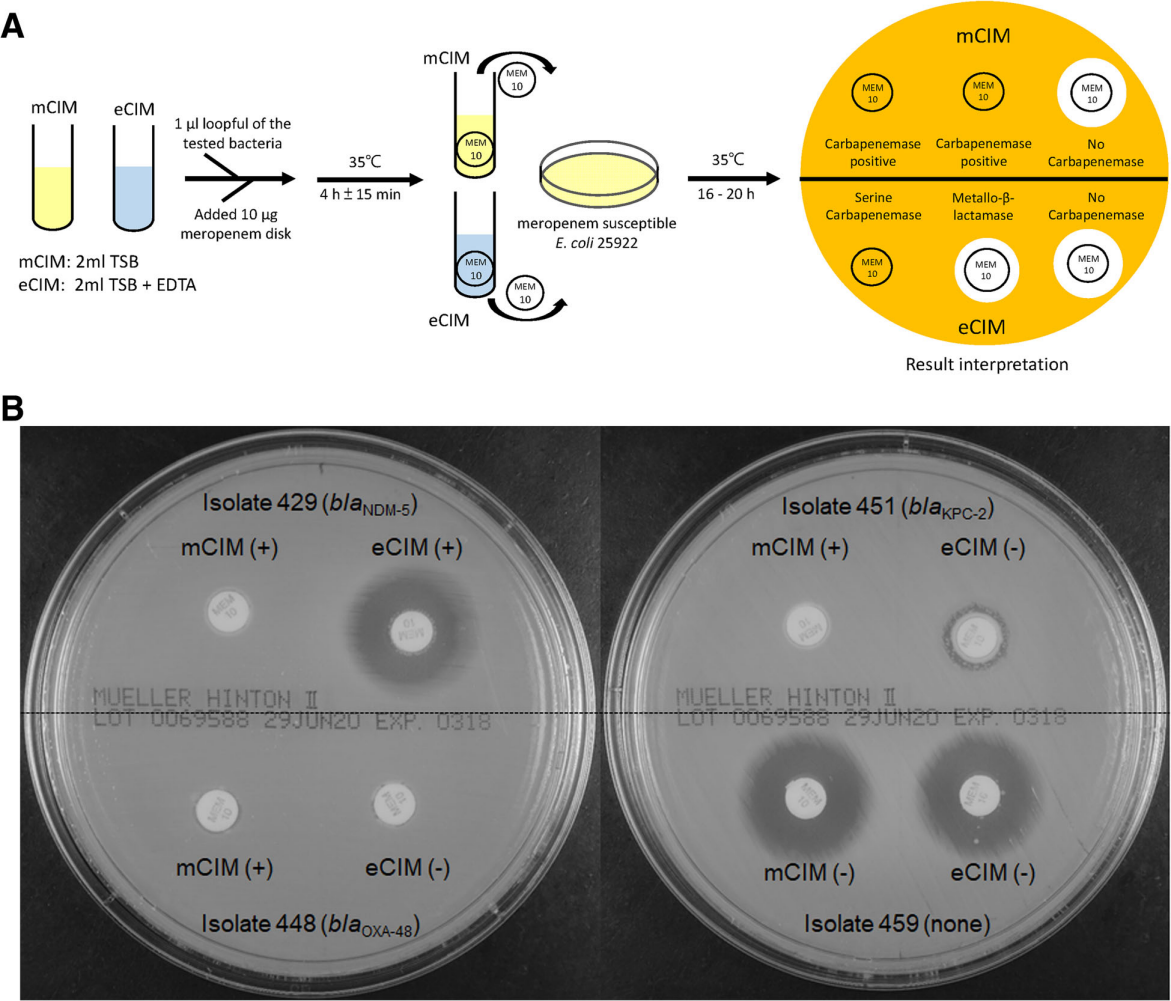
The procedure and interpretation of mCIM and eCIM. **a**. A 1-μL loopful of test CRE isolate is resuspended in two tubes containing 2 mL of TSB. One tube is devoid of EDTA (mCIM), while the other is supplemented with EDTA (eCIM). A meropenem (MEM) disk is submerged in each tube, and the tubes are incubated at 35 °C for 4 h ± 15 min. The disks are then removed from the tubes and placed on MH agar plates upon which a carbapenem-susceptible reporter *E. coli* ATCC 25922 has been freshly applied. The plates are incubated at 35 °C for 16 to 20 h before the zone sizes are recorded. **b**. Interpretation of mCIM and eCIM tests of 4 clinical *K. pneumoniae* isolates. Isolate 459 was carbapenemase negative; 429 had metallo-β-lactamase (*bla*_NDM-5_); 448 had serine carbapenemase (*bla*_OXA-48_); 451 had serine carbapenemase (*bla*_KPC-2_)

The mCIM and eCIM procedures and interpretation are shown in Fig. 1a. All CPE isolates and 90 non-CPE isolates were used to evaluate the mCIM and mCIM combined with eCIM (mCIM/eCIM) to detect carbapenemase producers. The interpretation of mCIM and eCIM results of clinical isolates were shown in Fig. 1b. Isolate 459 was a non-carbapenemase producer and isolates 448 (*bla*_OXA-48_ positive) and 451 (*bla*_KPC-2_ positive) were serine-carbapenemase producers (Fig. 1b). In contrast, isolate 429 expressed a metallo-carbapenemase (*bla*_NDM-5_ positive) and thus served as mCIM/eCIM positive control (Fig. 1b).

### Sensitivity and specificity of mCIM and eCIM for the detection of CPE

The distribution of carbapenemase genes and results of phenotypic detection of carbapenemases in our CRE isolates were shown in Table 2. The MHT, mCIM, and eCIM tests were replicated by two independent investigators to ensure reproducibility, and the results showed full reproducibility of these tests. Importantly, two isolates (1 *K. pneumoniae* and 1 *K. aerogenes*) with a false-positive result on MHT showed a negative result on mCIM. Moreover, 16 isolates with a false-negative result on MHT were mCIM positive (Table 2). The sensitivity and specificity for the mCIM to detect carbapenemase were both 100% in this study.

**Table 2:** Carbapenemase genes, MHT, mCIM, eCIM, and antibiotic susceptibility of 195 CRE isolates

Species	Carbapenemase-encoding genes^a^	No. of strains	MHT (n)		mCIM (n)		eCIM (n)		Carbapenem resistance (n)^b^			
			Neg	Pos	Neg	Pos	Neg	Pos	IPM	ETP	MEM	DOP
*Citrobacter koseri*	*bla*_OXA-48_	4	0	4	0	4	4	0	4	4	4	4
	None	3	3	0	3	0	ND	ND	2	3	2	2
*Enterobacter cloacae*	*bla*_IMP_	2	1	1	0	2	0	2	2	2	2	2
	*bla*_OXA-48_	1	0	1	0	1	1	0	0	1	1	0
	None	9	9	0	9	0	ND	ND	4	9	2	3
*Escherichia coli*	*bla*_KPC_	5	0	5	0	5	5	0	5	5	5	5
	*bla*_NDM_	11	6	5	0	11	0	11	11	11	11	11
	*bla*_OXA-48_	13	1	12	0	13	12	1	10	13	10	11
	*bla*_NDM_/*bla*_OXA-48_	3	1	2	0	3	2	1	3	3	3	3
	None	18	18	0	18	0	ND	ND	10	16	6	5
*Klebsiella aerogenes*	*bla*_VIM_	1	1	0	0	1	0	1	1	1	1	1
	*bla*_NDM_	1	0	1	0	1	0	1	1	1	1	1
	None	6	5	1	6	0	ND	ND	5	6	3	2
*Klebsiella pneumoniae*	*bla*_KPC_	29	0	29	0	29	29	0	29	29	29	29
	*bla*_IMP_	5	2	3	0	5	1	4	5	5	4	4
	*bla*_VIM_	2	1	1	0	2	0	2	2	2	2	2
	*bla*_NDM_	3	2	1	0	3	0	3	3	3	3	3
	*bla*_OXA-48_	23	1	22	0	23^**c**^	23	0	22	23	22	21
	*bla*_KPC_/*bla*_OXA-48_	2	0	2	0	2	2	0	2	2	2	2
	None	54	53	1	54	0	ND	ND	28	54	25	23
**Total**		**195**	**10**	**91**	**90**	**105**	**79**	**26**	**149**	**193**	**138**	**134**

^a^Class A carbapenemase, KPC; Class B carbapenemase, IMP, NDM, and VIM; Class D carbapenemase, OXA-48

^b^Antibiotic susceptibility to carbapenems was determined by disc diffusion. IPM, imipenem; ETP, ertapenem; MEM, meropenem; DOP, doripenem

^c^The results of 2 mCIM tests were interpreted as “indeterminate” (pinpoint colonies were present within a 16- to 18-mm zone)

*Pos* positive, *Neg* negative

The sensitivity for the mCIM combined with eCIM to detect different classes of carbapenemases was shown in Table 3. Twenty-five metallo-carbapenemase producers showed positive results by eCIM. However, a total of 3 isolates escaped the detection of metallo-carbapenemase, including 2 *E. coli* isolates having both *bla*_NDM_ and *bla*_OXA-48_, and 1 *K. pneumoniae* having *bla*_IMP_. One out of 77 isolates characterized to carry the serine carbapenemase genes showed positive results by eCIM (Table 3). The sensitivity of the eCIM observed in this study was 89.3% (25/28) and the specificity was 98.7% (76/77) as compared to the genotype (Table 3).

**Table 3:** Sensitivity and specificity for the mCIM combined with eCIM to detect different types of carbapenemase

mCIM + eCIM	***bla***_**OXA-48**_	***bla***_**KPC**_	***bla***_**NDM**_	***bla***_**IMP**_	***bla***_**VIM**_	***bla***_**NDM**_/***bla***_**OXA-48**_^**d**^	***bla***_**KPC**_/***bla***_**OXA-48**_	total
TP (n)	–	–	15	6	3	1	–	25
FN (n)	–	–	0	1	0	2	–	3
TN (n)	40^c^	34	–	–	–	–	2	76
FP (n)	1	0	–	–	–	–	0	1
Sensitivity (%)^a^	–	–	100	85.7	100	33.3	–	89.3
Specificity (%)^b^	97.6	100	–	–	–	–	100	98.7

^a^Sensitivity = TP/TP + FN

^b^Specificity = TN/TN + FP

^c^The results of 2 mCIM tests were interpreted as “indeterminate” (pinpoint colonies were present within a 16- to 18-mm zone)

^d^Isolates with *bla*_NDM_/*bla*_OXA-48_ were defined as metallo-carbapenemase positive

*TP* true positive, *FN* false negative

mCIM/eCIM assay is designed to simultaneously detect and distinguish the types of carbapenemase. Sfeir et al. showed that the sensitivity and specificity of eCIM was both 100%, in the presence of 5 mM EDTA [21]. Our results showed that the sensitivity and specificity for the mCIM/eCIM to detect MBLs is 89.3 and 98.7%, respectively (Table 3). *K. pneumoniae* isolate 456, an IMP-8 producer, showed a false-negative result by mCIM/eCIM (Table 4). Our sequencing data identified a wild-type *bla*_IMP-8_ in isolate 456. However, isolate 456 showed low resistance to carbapenems (Table 4). Minimum inhibitory concentrations (MICs) of isolate 456 to imipenem, ertapenem, and meropenem were 0.5, 2, and ≤ 0.25, respectively (Table 4). *E. coli* isolates 488 and 492 containing *bla*_OXA-48_/*bla*_NDM-5_ also showed false-negative results by mCIM/eCIM (Table 4).

**Table 4:** Characteristics of isolates with false mCIM/eCIM results

Isolate^**a**^	Carbapenemase	MIC (μg/ml)			Disc zone (mm)						Phenotypic detection	
		IPM	ETP	MEM	IPM	ETP	MEM	DOP	mCIM	eCIM	MHT	mCIM/eCIM^a^
**False-positive**												
*E. coli* 514	*bla*_OXA-48_	8	≥ 8	≥ 16	16	12	15	16	6	23	–	+
**False-negative**												
*K. pneumoniae* 456	*bla*_IMP-8_	0.5	2	≤0.25	22	18	25	25	19	20	–	–
*E. coli* 488	*bla*_OXA-48_/*bla*_NDM-5_	≥ 16	≥ 8	8	6	6	6	6	6	6	+	–
*E. coli* 492	*bla*_OXA-48_/*bla*_NDM-5_	≥ 16	≥ 8	≥ 16	6	6	6	6	6	6	+	–

^a^An isolate is positive for metallo-carbapenemase production when the eCIM zone size increases by ≥5 mm compared to the zone size observed for the mCIM and is considered negative for a metallo-carbapenemase if the increase in zone size is < 4 mm

*IPM* imipenem, *ETP* ertapenem, *MEM* meropenem

## Discussion

The first CLSI recommended growth-based carbapenemase detection test was the MHT in 2009. Here, we showed that MHT had excellent sensitivity for the detection of *bla*_OXA-48_ (95.1%) and *bla*_KPC_ (100%) (non-metallo-carbapenemase genes) (Table 1). However, the MHT has some limitations, notably insensitivity for the detection of MBL enzymes [22]. Our results (Table 1) are consistent with previous studies which showed a high rate of false-negative MHT results with MBL-producers [19, 23, 24]. Moreover, MHT results are often difficult to interpret, and false-positive results are observed for isolates producing ESBL or AmpC β-lactamase with porin loss [25].

The overall sensitivity and specificity of MHT in this study for the detection of CPE was 84.8 and 97.8%, respectively (Table 1). However, 16 isolates with a false-negative result on MHT were mCIM positive (Table 2)*.* Our results showed that the sensitivity of mCIM to detect MBLs is 100% and the mCIM is more accurate compared to MHT. mCIM/eCIM assay is designed to simultaneously detect and distinguish the different types of carbapenemase. Previous studies showed that the sensitivity and specificity of eCIM was both 100%, in the presence of 5 mM EDTA [21]. However, the number of *E. coli* and *K. pneumoniae* isolates used to evaluate mCIM/eCIM assay for identifying CPE in Sfeir’s report was limited [21]. In this study, 50 *E. coli* (32 carbapenemase producers) and 118 *K. pneumoniae* (64 carbapenemase producers) were enrolled to evaluate mCIM/eCIM for detecting CPE*.* Our results showed that the sensitivity and specificity for the mCIM/eCIM to detect MBLs is 89.3 and 98.7%, respectively (Table 3). Therefore, the inconsistency of sensitivity and specificity for the mCIM/eCIM assay to detect MBLs between our results and Sfeir’s findings might be caused by the number of tested isolates. In this study, we used PCR targeting carbapenemase genes as the gold standard to evaluate the performance of phenotypic tests for identifying carbapenemase producers. This might be a limitation as new or truncated carbapenemase genes might not be identified consistently with the phenotype.

*K. pneumoniae* isolate 456 (*bla*_IMP-8_ positive) showed a false-negative result by mCIM/eCIM (Table 4). Although wild-type *bla*_IMP-8_ was identified in isolate 456, the expression level of *bla*_IMP-8_ in isolate 456 remains to be determined. Isolate 456 showed low resistance to carbapenems (Table 4). In addition, no additional carbapenemase genes (*bla*_GES_, *bla*_IMI_, *bla*_SME_, *bla*_SPM_, *bla*_SIM_, *bla*_DIM_, and *bla*_GIM_) were detected in isolate 456. Therefore, it is worth investigating whether the carbapenem resistance level of bacteria is associated with the accuracy of mCIM/eCIM to detect carbapenemase. *E. coli* isolates 488 and 492 having *bla*_OXA-48_/*bla*_NDM-5_ showed false-negative results for MBL detection by mCIM/eCIM (Table 4). These results suggest the expression of both carbapenemase genes can cause the misidentification of MBLs by mCIM/eCIM. *E. coli* isolate 514, which carried only the serine carbapenemase genes (*bla*_OXA-48_), showed false-positive results by eCIM based on the PCR targeting carbapenemase genes as the gold standard in this study (Table 4). However, we could not rule out the possibility of a novel carbapenemase gene in isolate 514, and therefore, an entire genome analysis of isolate 514 is worth investigating in the future.

## Conclusion

Understanding the mechanism(s) causing carbapenem resistance of *Enterobacteriaceae* has important clinical implications and results in different prevention measurements and individualized antibiotic therapy. In this study, our results indicate that the phenotypic detection, mCIM combined with eCIM, showed high sensitivity and specificity to detect carbapenemase-producing *Enterobacteriaceae*, compared with MHT.

## Methods

### Identification of carbapenem-resistant *Enterobacteriaceae* isolates

*Enterobacteriaceae* isolates were recovered in En Chu Kong hospital, 2011 to 2019. These isolates were identified in the clinical laboratory by colony morphology, Gram stain, biochemical tests, and the Vitek 2 system (bioMérieux, Marcy l′Etoile, France) according to the manufacturer’s recommendations. Susceptibility to third-generation cephalosporins (ceftazidime or ceftriaxone, 30 μg/disc, BD BBL™ Sensi-Disc™, Sparks, MD, USA) for *Enterobacteriaceae* isolates were determined by the disk diffusion method on Mueller-Hinton (MH) agar plates (Bio-Rad, Marne la Coquette, France) based on the CLSI guidelines (M100-S30) [26]. Third-generation cephalosporin -resistant isolates were further tested for their susceptibility to carbapenems, including imipenem, ertapenem, meropenem, and doripenem (10 μg/disc, BD BBL™, USA). MICs to imipenem, ertapenem, and meropenem were further determined by Vitek 2 using the AST-N322 card according to the manufacturer’s instructions. A total of 419 CRE isolates were identified and stored at − 80 °C in tryptic soy broth (TSB) containing 20% glycerol (v/v) until used.

### Carbapenemase gene detection

Bacterial genomic DNA was isolated from bacteria grown overnight at 37 °C in 3 mL LB broth. Bacterial culture was centrifuged for 1 min at 12,000 rpm, and the supernatant was removed. Crude DNA extracts were obtained by suspending the pellet in 300 μL distilled water and boiling at 95 °C for 10 min, followed by centrifugation at 12,000 rpm for 5 min. The supernatant containing DNA was transferred to a new eppendorf tube, and the DNA samples were stored at 4 °C until testing.

PCR targeting carbapenemase genes was used as the standard to assess the performance of phenotypic tests. Therefore, PCR amplification for the detection of β-lactamase genes (*bla*_KPC,_ *bla*_NDM,_ *bla*_IMP,_ *bla*_VIM,_ *bla*_OXA-48,_ *bla*_GES_, *bla*_IMI_, *bla*_SME_, *bla*_SPM_, *bla*_SIM_, *bla*_DIM_, and *bla*_GIM_) was carried out on a iCycler iQ5 real time PCR system (Bio-Rad, USA) with the HotStar PCR SuperMix (GeneDireX, USA). Primers and PCR procedures used in β-lactamase genes detection were described in previous studies [15, 27–29]. The PCR products were analyzed by electrophoresis with 2% agarose gels in 0.5× Tris-borate-EDTA (TBE) buffer. The gels were stained with Novel Juice (GeneDireX, USA), and the PCR products were visualized with UV light. Clinical *K. pneumoniae* isolates harboring *bla*_KPC,_ *bla*_NDM,_ *bla*_IMP,_ *bla*_VIM,_ and *bla*_OXA-48_ were used as PCR positive controls. In accessibility to have strains with *bla*_GES_, *bla*_IMI_, *bla*_SME_, *bla*_SPM_, *bla*_SIM_, *bla*_DIM_, or *bla*_GIM_ as our PCR controls. The PCR products with relevant expected size were purified and verified by sequencing.

### Phenotypic detection of carbapenemase-producing *Enterobacteriaceae*

MHT, mCIM, and eCIM were performed on CRE isolates according to the CLSI guidelines to detect the presence of carbapenemase [20]. The mCIM and eCIM procedures are illustrated in Fig. 1a. In brief, a 1-μL loopful of bacteria was resuspended in a 2-mL tube of TSB. Another 1-μL loopful of bacteria was resuspended in a 2-mL tube of TSB supplemented with EDTA (Thermo Fisher Scientific, Carlsbad, CA, USA) at a final concentration of 5 mM (add 20 μL of 0.5 M EDTA to 2 mL of TSB). A meropenem disk was placed in each tube, and the tubes were incubated at 35 °C for 4 h ± 15 min. Subsequently, the disks were removed and applied to MH agar plates freshly plated with a 0.5 McFarland suspension of a carbapenem-susceptible *E. coli* ATCC 25922 strain (Fig. 1a). The plates were incubated at 35 °C for 16 to 20 h and the mCIM and eCIM results were interpreted as previously described [21, 30]. The mCIM is considered negative if the zone size is ≥19 mm, positive if the zone size is 6 to 15 mm, or intermediate (defined as positive) if pinpoint colonies are present within a 16- to 18-mm zone [21, 30]. An isolate is positive for metallo-carbapenemase production when the eCIM zone size increases by ≥5 mm compared to the zone size observed for the mCIM and is considered negative for a metallo-carbapenemase if the increase in zone size is < 4 mm [21, 30]. According to CLSI guidelines [20], *K. pneumoniae* ATCC BAA-1706 (carbapenemase negative), *K. pneumoniae* ATCC BAA-1705 (*bla*_KPC_ positive), and *K. pneumoniae* ATCC BAA-2146 (*bla*_NDM_ positive) were used as internal controls for mCIM and eCIM tests. The MHT, mCIM and eCIM tests were replicated by two independent investigators to ensure reproducibility.

## Data Availability

All data generated or analyzed during this study are included in this published article.

## Abbreviations

CLSI: Clinical & Laboratory Standards Institute
CPE: Carbapenemase-producing *Enterobacteriaceae*
CRE: Carbapenemase-resistant *Enterobacteriaceae*
DOP: Doripenem
eCIM: EDTA carbapenem inactivation method
ETP: Ertapenem
FN: False negative
FP: False positive
IPM: Imipenem
MBL: Metallo-β-lactamases
MEM: Meropenem
mCIM: Modified carbapenem inactivation method
MHT: Modified Hodge test
MIC: Minimum inhibitory concentration
TN: True negative
TP: True positive
